# Elucidation of the relationship between masticatory muscle activity and masticatory movement. Report 1. Mechanism of occlusal force generation

**DOI:** 10.1002/cre2.725

**Published:** 2023-03-15

**Authors:** Katsumi Niwa, Nobuo Takahashi

**Affiliations:** ^1^ Division of Dental Radiology, Department of Diagnostic and Therapeutic Sciences Meikai University School of Dentistry Saitama Japan

**Keywords:** bite force, contraction force of masticatory muscle, masticatory muscle activity, occlusal force

## Abstract

**Objectives:**

The purpose of this study was to elucidate how masticatory muscles are involved in the generation of occlusal force.

**Materials and Methods:**

The experiment was conducted by fabricating an experimental apparatus for a unilateral occlusion model with the masticatory muscles imparted. The experimental apparatus was fabricated by enlarging the lateral photograph of a dried adult skull specimen 3.5 times larger than that of a standard adult and drawing the outlines of the maxilla and mandible, canines and molars of the upper and lower jaws, and temporomandibular joint on a wooden board. The masticatory muscles used in the experiment were the masseter muscle, the temporalis muscle (anterior and posterior muscle bundles), and the lateral pterygoid muscle. For the measurement of the contractile force of the masticatory muscle, we used the spring scale. For the food, we used cut plastic cylinders.

**Results:**

The results of the experiment revealed the following: First, the occlusal force was generated under the condition that the contraction forces of all the masticatory muscles were balanced. Second, when the occlusal force was applied to food, the occlusal planes of the upper and lower jaws were parallel. Third, the occlusal force occurred perpendicular to the occlusal plane. Fourth, the occlusal force was generated with a force greater than the contraction force of the individual masticatory muscles. And finally, even if occlusal force was applied to the food, the occlusal force did not load the temporomandibular joint.

**Conclusion:**

Occlusal force is not generated by the action of a single masticatory muscle but under the balanced contractile force of all masticatory muscles. The occlusal force then emerges with a force greater than the contraction force of all the masticatory muscles, and its direction occurs perpendicular to the occlusal plane.

## 1 INTRODUCTION

The relationship between masticatory muscle activity and masticatory movement is generally described in many textbooks (Bell, 1986; Ogawa & Sasaki, 2002; Okeson, 2003a; Slavicek, 2006). Okeson also describes this relationship as follows. Masticatory movement is a highly systematized and complex neuromuscular activity (Okeson, 2003a). However, this description does not allow us to understand how the jaw moves during mastication. Ramfjord and Ash (1983) have the following to say about this: With today's knowledge, it is impossible to completely analyze the various masticatory movements that are associated with any jaw movement. This statement indicates that the relationship between masticatory muscle activity and masticatory movement is unresolved even today. However, the movement of the jaw during mastication is one of the most important and fundamental issues in dentistry that must be clarified.

Aside from the movement of the jaw during mastication, the relationship between the function of individual masticatory muscles and the movement of the jaw is described in many textbooks. For example, the masseter muscle is described as responsible for pulling up the mandible and generating occlusal force (Bell, 1986; Ogawa & Sasaki, 2002; Okeson, 2003b; Ramfjord & Ash, 1983; Slavicek, 2006). Okeson ( 2003b) describes the relationship between masseter muscle activity and mandibular movement as follows. As the fibers of the masseter contract, the mandible is elevated and the teeth are brought into contact. However, for the mandible to move in this manner, the condyle of the temporomandibular joint (TMJ) must act as a fulcrum in the principle of leverage. When the upper and lower molars chew food, the condyle moves to an anteroinferior position along the anterior wall of the mandibular fossa. The amount of movement depends on the size of the food. Moreover, the condyle is free to move. This means that the condyle cannot act as a fulcrum during food crushing. Therefore, when crushing food, the contractile force of the masseter muscle can pull up the ramus of the mandible but cannot bring the teeth into contact.

Next, let's consider the problem that arises when chewing food with the mandibular first molar. The textbook (Kamijo, 1965b) describes the attachment position of the masseter muscle and the running of the fibers as follows. The masseter muscle originates from the inferior border of the zygomatic arch and attaches to almost the entire external surface of the ramus of the mandible. The shallow muscle bundles of the masseter muscle run obliquely upward, while the deep muscle bundles run vertically. Furthermore, the ramus of the mandible is located between the first molar and the TMJ. What can be inferred from these descriptions is that when the contraction force of the masseter muscle is exerted, part of that force acts on the TMJ as well as the first molar. Furthermore, the direction of contraction of the masseter muscle as a whole is estimated to be closer to vertical than the direction of contraction of the shallow muscle bundle but still directed anteriorly and upward. This means that the occlusal force applied to the food is applied anteriorly upward, not vertically from the occlusal plane. This application of occlusal forces to the food is both inefficient and unnatural. All of this makes it hard to believe that occlusal forces are generated by the contractile force of the masseter muscles alone. Even for other masticatory muscles, the textbooks describe many movements that are mechanically impossible (Bell, 1986; Kamijo, 1965a; Körber, 1975; Ogawa & Sasaki, 2002; Okeson, 2003a; Ramfjord & Ash, 1983; Slavicek, 2006).

Hesse and colleagues (1988) describe the relationship between the occlusal force and food as follows. To maintain the static equilibrium of the mandible, the occlusal force generated from the contractile force of the masticatory muscles during mastication must always pass through the occlusal points. Otherwise, the mandible will rotate under the action of this force. This statement indicates that not only the masseter muscle but also other masticatory muscles must be involved in the generation of the occlusal force. There are reports on the measurement of human bite force and the bite force of individual teeth by Gibbs et al. (1981, 1986) and Hattori et al. (1996). However, there is no description of how the occlusal force is generated.

The authors have created an experimental apparatus for a unilateral occlusion model equipped with a mechanism to change the contractile force of the three masticatory muscles (masseter, temporalis, and lateral pterygoid). Using this apparatus, we have been conducting experiments to clarify the relationship between masticatory muscle activity and masticatory movement. This is the first report of the study, which clarifies the mechanism of occlusal force generation.

## 2 MATERIALS AND METHODS

**Figure 1:**
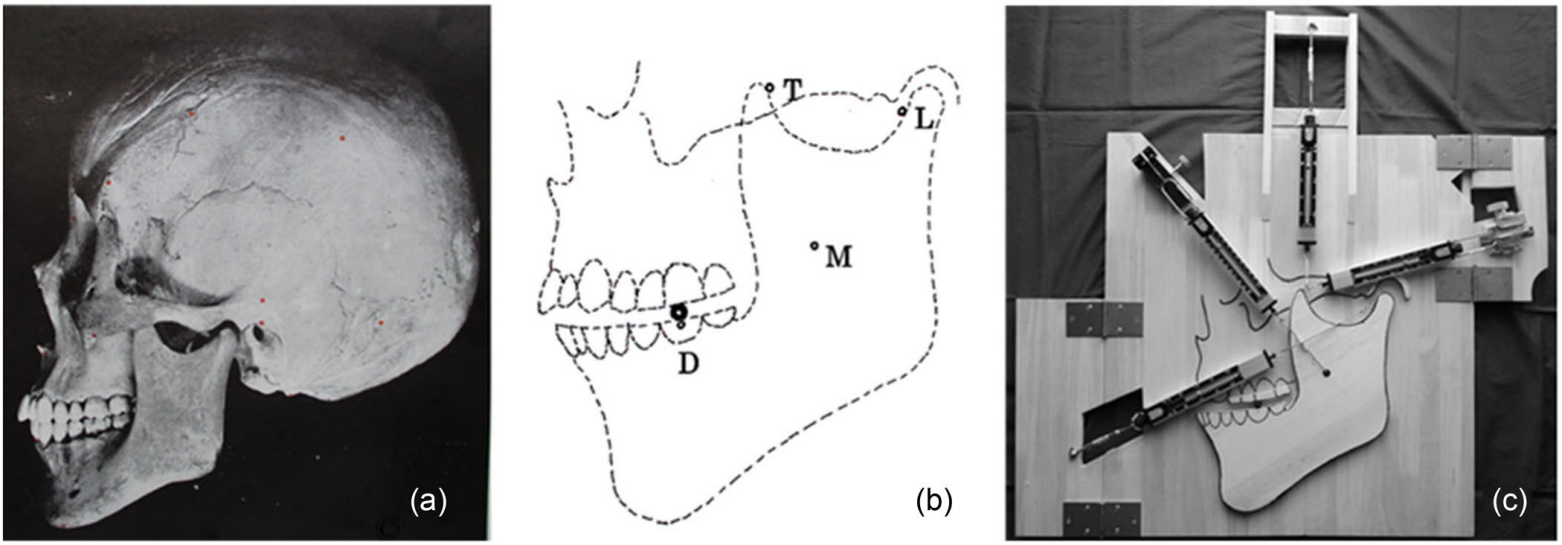
Lateral photograph of a dried skull specimen of a modern human and its trace image and the experimental apparatus of the unilateral occlusion model. (a) Lateral photograph of a dried skull specimen of a modern human borrowed from the Kamijo literature. (b) Traced image of Figure 1 (a). (c) Experimental apparatus of a unilateral occlusion model.

A lateral photograph of a dried skull specimen of a modern human, borrowed from the textbook of Kamijo (1965b) is shown in Figure 1a. A traced diagram of this is shown in Figure 1b. This figure was enlarged to 3.5 times the average size of an adult (Kamijo, 1965b) and drawn on a 1 cm thick wooden board to create the experimental apparatus of a unilateral occlusion model. The occlusal surface of the maxillary posterior teeth was cut from another 1 cm thick wooden board and glued to the position on the trace of the apparatus. The mandible was cut out from a 1 cm thick paulownia plate.

The occlusal surfaces of the maxilla and mandible were cut to present a right angle to the respective wooden planks. In other words, the occlusal surfaces were fabricated to be perpendicular to the wooden board of the experimental apparatus. The occlusal plane was designed to be horizontal when viewed from the frontal plane and to have a Spee curve (Dawson, 1974) when viewed from the sagittal plane, as the flat occlusal plane by occlusal wear as seen in Leigh's (1929) and other reports (Beyron, 1964; Hioki, 1979). The Spee curve was obtained from Figure 1a as a curve connecting the midpoints of the cusp tips of the upper and lower posterior teeth.

For the temporomandibular joint, the inner wall of the mandibular fossa was made from a 1 cm thick wooden plate and glued to the apparatus. In the centric occlusion, the condyle was positioned in the center of the mandibular fossa. For the food, we used a cut plastic cylinder 1 cm in diameter. The food corresponded to a size of 2.8 mm in the oral cavity. This size was chosen because it was determined that it could apply a large occlusal force when the food was very hard. Because of the cylindrical shape of the food, the food rolls freely on the flat occlusal surface when a slight force is applied from the proximal or distal direction.

In Figure 1b, the small rings on the mandible are the measurement points set to measure the contraction force of the masticatory muscle. Point M is the masseter muscle, point T is the temporalis muscle, and point L is the lateral pterygoid muscle. These points were determined based on the moving end of each muscle. The M‐point of the masseter muscle, which is particularly difficult to determine, was set at the center of the uppermost part of the adhesion rough surface of the masseter muscle found on the ramus of the mandible, referring to the textbook of Kamijo (1965b). This M‐point also serves as a measurement of the contraction force of the medial pterygoid muscle. The reason for this is that the masseter muscle and the medial pterygoid muscle have only a slight difference in the direction of contraction on the two‐dimensional plane (Kamijo, 1965b) and therefore, within the scope of the purpose of this experiment, these muscles are considered to work almost identically. The black bold ring on the occlusal surface of the mandibular first molar indicates the position of the food. Point D, directly below the occlusal surface of the mandibular first molar, is the measurement point for occlusal force.

A small metal ring (9 mm outer diameter, 5.5 mm inner diameter) was attached to each measurement point on the mandible, and one end of the spring scale was attached to it. The other end of the scale was fixed in such a way that the direction of the scale coincided with the direction of contraction of the masticatory muscle. In addition, a rack and pinion or turnbuckle was attached to the other end of the scale. By manipulating these, the contractile force of the individual masticatory muscles was made to change. The masticatory muscles set up for the experiment were the masseter muscle, the anterior and posterior muscle bundles of the temporalis muscle, and the lateral pterygoid muscle. The experimental apparatus for the unilateral occlusion model is shown in Figure 1c.

The experiment was conducted according to the following procedure. First, we applied moderate contractile force to each masticatory muscle and adjusted the contractile force of each muscle so that the articulation of the upper and lower molars was stabilized in centric occlusion. Second, we placed the cylindrical food on the center of the occlusal surface of the mandibular first molar and adjusted the contractile force of each masticatory muscle again so that the food would not move from that position. Third, after the condition in the second step was satisfied, the contractile force of each masticatory muscle was adjusted so that the condyle was stabilized in the anterior inferior position of the mandibular fossa, about 3 mm away from the anterior wall. Fourth, after the condition in the third step was met, the contraction force of each masticatory muscle was measured. Finally, the occlusal force applied to the food was measured.

## 3 RESULTS

### 3.1 Experiment 1: Stillness of the food is established when the food is chewed and the parallelism of the occlusal planes of the upper and lower jaws is attained

When the food is chewed and a biting force is applied, the food becomes motionless and stationary on the occlusal surface of the mandibular first molar. Therefore, the primary purpose of this experiment was to elucidate the reason why the food is stationary, and the second was to clarify the relationship between the upper and lower occlusal planes when the food is chewed.

The experiment was conducted under the following conditions. First, the contraction force of the masseter muscle was set at 14.7 N (1.5 kgf). The contraction force of the masseter muscle was set at 14.7 N for the following reasons: Basic experiments have shown that when food is at rest on the first molar, the ratio of the contractile forces of the individual masticatory muscles is the same, and because the measurement limit of the spring scale is as small as several kilograms, we thought that 14.7 N would be optimal for reproducible measurements. Second, the food was placed on the center of the occlusal surface of the mandibular first molar. Third, the contractile force of the masticatory muscles other than the masseter muscle was adjusted to prevent the food from moving from that position. Finally, the contractile force of the masticatory muscles was adjusted so that the condyle was stabilized at the anterior‐inferior position of the anterior wall of the mandibular fossa, about 3 mm away from the anterior wall without contact.

**Figure 2:**
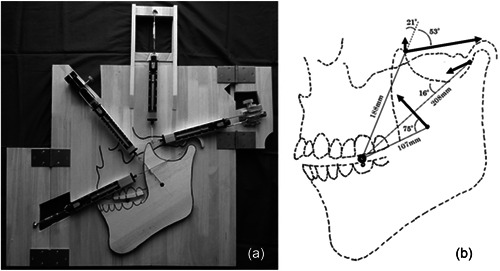
Static state of food established by the equilibrium of the contraction force of the masticatory muscles. (a) When the contraction force of all the masticatory muscles is balanced by chewing the food, the food becomes motionless and stationary. The photograph shows the food at rest when it is chewed. (b) The measured contraction force of each masticatory muscle is displayed as a vector when the food is in a stationary state. It also connects the center of the food and the starting point of the vector by a straight line and displays the distance between them.

When the third condition above was met, the mandible would chew the food and not move. The stationary state of the food is shown in Figure 2a. Figure 2a also shows that the food is resting on the occlusal surface of the mandibular first molar, and the occlusal planes of the upper and lower jaws are parallel. In this resting state, the contact between the wooden plate of the experimental apparatus and the mandible was found to be only at one point of the chin area, while the other parts were slightly floating. Therefore, the coefficient of friction between the two is considered to be close to zero.

First, we considered the reason for chewing food and remaining stationary from the perspective of occlusal mechanics. When the stationary state was established, the measured contraction force of the masticatory muscles was as follows: The masseter muscle was 14.7 N, the anterior bundle of the temporalis muscle was 4.0 N, the posterior bundle was 15.7 N, and the lateral pterygoid muscle was 8.8 N. Figure 2b shows the contraction force of each masticatory muscle as a vector with a bold arrow. In addition, the center of the food and the starting point of the vector is connected by a straight line, and its distance on the experimental apparatus is shown.

Looking at the direction of the vectors as shown in Figure 2b, we can see that the contraction forces of the masseter muscle, the anterior bundle of the temporalis muscle, and the lateral pterygoid muscle act as a force to rotate the mandible counterclockwise around the food. On the other hand, the posterior bundle of the temporalis muscle acts as a clockwise rotational force. It is thought that the state in which food comes to rest is due to the equilibrium of these two conflicting rotational forces. The equilibrium of the two conflicting rotational forces can be proved by the moment of force.

Before the calculation, the moments of the contraction force of the masseter muscle, the anterior and posterior bundles of the temporalis muscle, and the lateral pterygoid muscle are abbreviated as M‐M (Moment of Masseter muscle's contraction force), M‐TF, M‐TP, and M‐L, respectively. The moment of each masticatory muscle was calculated as shown in Equation (1) below.

M‐M=14.7×sin75×0.107=1.52[Nm]

M‐TF=4.0×sin22×0.188=0.28

M‐TP=15.7×sin52×0.188=2.33

(1) M‐L=8.8×sin16×0.208=0.51

The angle between the vector and the line was measured directly from the experimental setup.

Next, we sought to find the sum of all the moments. In calculating the sum of all the moments, we let M‐M, M‐TF, and M‐L in the counterclockwise direction be negative values and M‐TP be a positive value. Substituting the numbers in Equation (1) into the left‐hand side of Equation (2) leads to the result on the right‐hand side.

(2) (−M‐M)+(−M‐TF)+(−M‐L)+(+M‐TP)=+0.02[Nm].

The sum of all moments is +0.02Nm. 0.02 Nm is equivalent to about 36 gf in terms of the contractile force of the lateral pterygoid muscle. This value can be thought to be within the error range, considering that the experimental setup is two‐dimensional and taking into account the poor accuracy of the spring scale (described in detail in the discussion). In other words, the sum of the moments of the contraction force of all the masticatory muscles is zero. This result shows that when the moments of the contraction forces of all the masticatory muscles are balanced with the food as the center of rotation, the food will not move.

**Figure 3:**
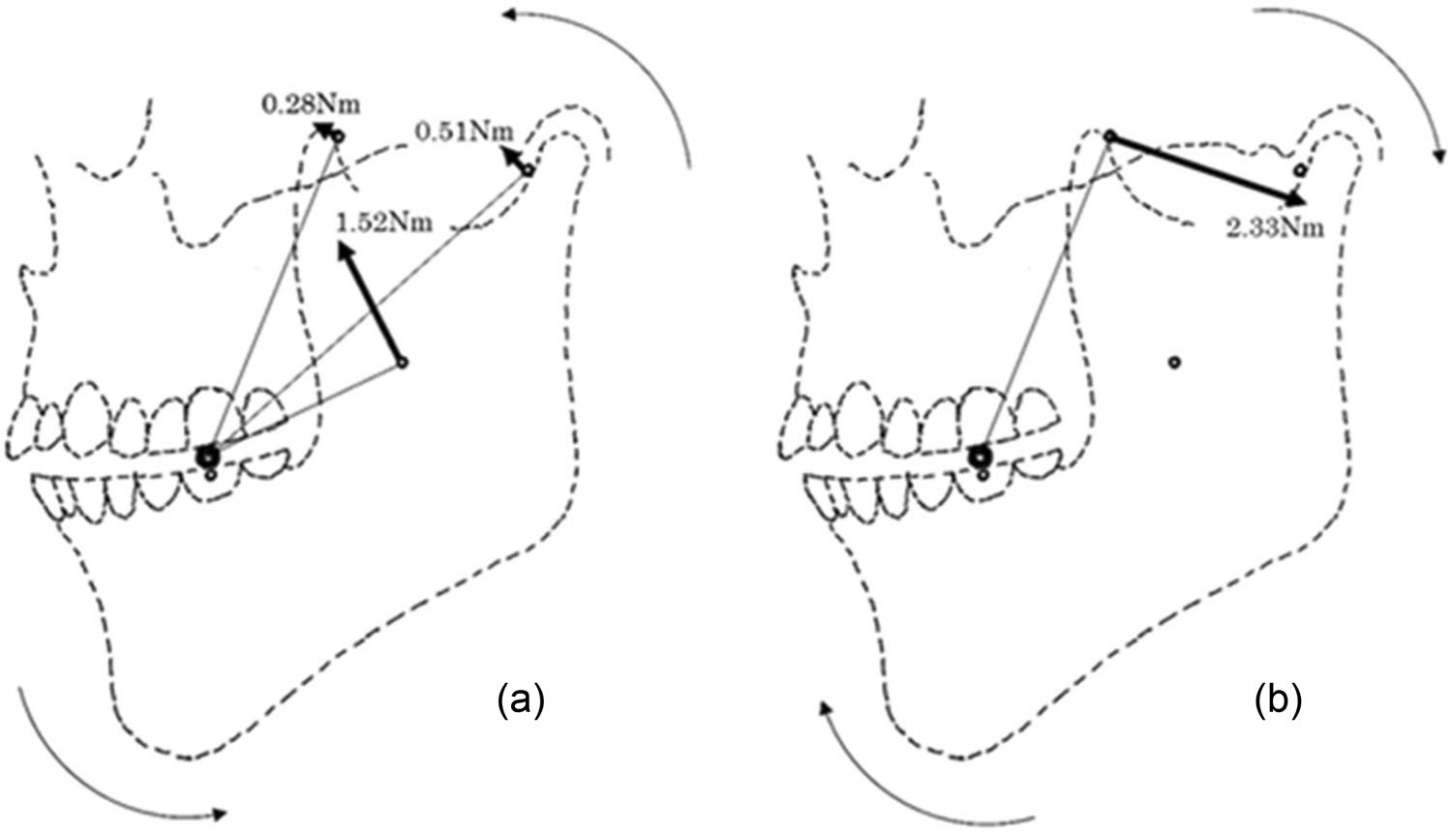
Parallelism of the occlusal planes of the upper and lower jaws established by the equilibrium of the contraction force of the masticatory muscles. (a) The counterclockwise moment due to the contraction force of the masseter muscle, the anterior bundle of the temporalis muscle, and the lateral pterygoid muscle is shown. (b) The clockwise moment due to the posterior bundle of the temporalis muscle is shown.

Next, we considered the relationship between the occlusal planes of the upper and lower jaws when chewing food. The moments of the masticatory muscles obtained from the previous experiment are shown in Figure 3a,b, divided into two different directions of rotation. In the previous experiment, we found that when two conflicting rotational forces were balanced, the food remained stationary without moving. If the equilibrium between the two rotational forces is broken and one rotational force is greater or less than the other, an angle will be created between the occlusal planes of the upper and lower jaws with the food as the center of rotation. This causes a proximal or distal force to act upon the food, causing the cylindrical food to move on the occlusal plane. The reason why the food does not move when the food is chewed is because the occlusal planes of the upper and lower jaws are parallel. The occlusal significance of the parallelism of the occlusal surfaces of the upper and lower jaws is that the occlusal force is applied vertically through the food from the occlusal surface of the mandible to the occlusal surface of the maxilla. This would satisfy the requirement that the occlusal force pass through the occlusal point as reported by Hesse and Naeije (1988). Furthermore, we found something interesting in this experiment. That is, the occlusal force is generated on the food in this static state of the mandible. Let us now consider the movement of the jaw during masticatory movements. If the food is soft, the mandible can crush it even as it moves. However, the harder the food is, the more occlusal force required to crush it occurs with the mandible at rest. The occlusal forces applied to the food with the mandible at rest will satisfy Hesse's conditions.

The results of Experiment 1 can be summarized as follows: First, when the food was chewed and the contraction force of all the masticatory muscles was balanced, the food was motionless and still. Second, when the food was stationary, the occlusal planes of the upper and lower jaws were parallel. Finally, when the food was stationary, the occlusal force was applied to the food.

### 3.2 Experiment 2: Measurement of occlusal force and its characteristics

The purpose of this experiment is to measure the magnitude of the occlusal force, derive it theoretically, and characterize it. Before measuring the occlusal force, it is necessary to determine the direction of the scale to measure the occlusal force accurately. The direction of the scale was determined as follows. With the food chewed and held stationary, we measured the occlusal force by changing the direction of the spring scale around point D in various ways. We then found the direction in which the food loosened slightly with the least amount of traction and determined that direction as the direction of measurement. “Slightly loose” refers to the smallest gap between the upper and lower occlusal planes where the circular food can rotate freely. When measuring the occlusal force, gradually increasing the pulling force of the occlusal force measuring scale will slightly reduce the force applied to food without changing the contraction force of all masticatory muscles. The food then loosens slightly in the occlusal planes of the upper and lower jaws. The strength of the pull at that time was determined as the occlusal force.

**Figure 4:**
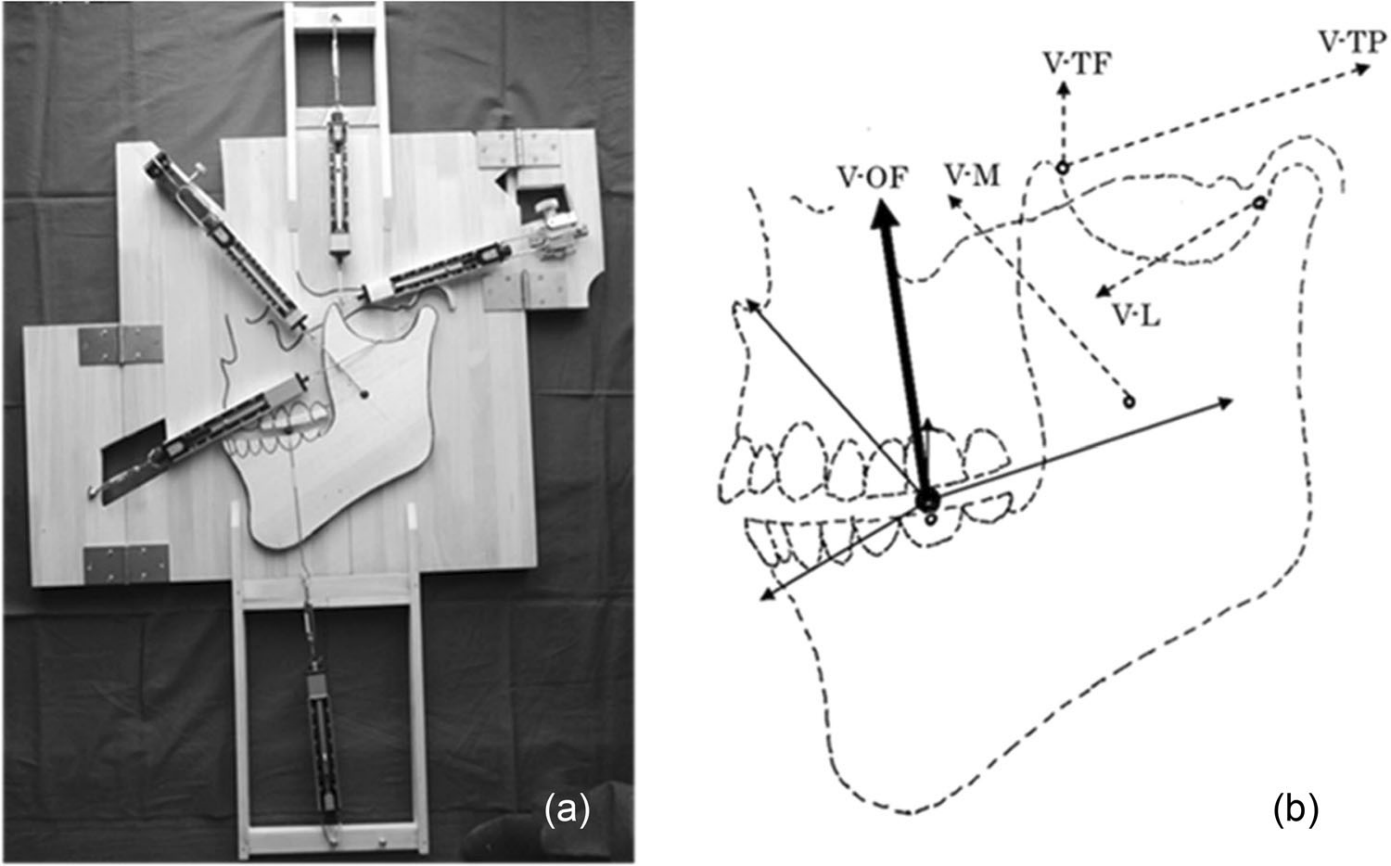
Magnitude and direction of the occlusal force occurring under the equilibrium of the contraction force of all the masticatory muscles. (a) The photograph shows a balanced state of the contraction force of all the masticatory muscles. (b) The contraction force of each masticatory muscle is represented by a vector. The contraction force of the masseter muscle is V‐M, the anterior bundle of the temporalis muscle is V‐TF, the posterior bundle of the temporalis muscle is V‐TP, and the lateral pterygoid muscle is V‐L. They are indicated by dotted arrows.

In the experiment of measuring occlusal force, Figure 4a shows the state in which the food was at rest due to the balanced contraction force of masticatory muscles. The masseter muscle contraction force measured in this experiment was 14.7 N, the anterior temporalis muscle bundle was 4.6 N, the posterior temporalis muscle bundle was 16.7 N, the lateral pterygoid muscle was 9.2 N, and the occlusal force was 15.68 N.

Here, a question comes to mind. The question is why the contraction force of the masseter muscle was the same in Experiments 1 and 2, but the contraction force of the other masticatory muscles was different. The reason for this is that the position of the condyle in relation to the anterior wall of the mandibular fossa was not the same in the two experiments. Previous experiments have shown that even if the contraction force of the masseter muscle is the same if the position of the condyle is slightly different, the contraction force of the other masticatory muscles to hold the food stationary will be different.

First, we calculated the occlusal force generated from the balanced contraction force of the masticatory muscles theoretically. The contraction force of the masseter muscle, anterior muscle bundle, posterior muscle bundle, and lateral pterygoid muscle is named V‐M (Vector–Masseter's contraction force), V‐TF, V‐TP, and V‐L, respectively. In Figure 4b, each vector is indicated by a dotted arrow. These vectors have different start points and directions. Therefore, we moved the vector so that the starting point of each vector would coincide with the center of the food. The vector after the move is shown with a solid arrow in Figure 4b. Next, we determined the sum of the vectors of all masticatory muscles, as shown on the left‐hand side of Equation (3).

(3) (V‐M)+(V‐TF)+(V‐TP)+(V‐L)=(newvector).

Calculating the vector sum led to a new vector as shown in the right‐hand side of Equation (3). We call this new vector Vector‐OF (V‐OF). When V‐OF is calculated, 15.88 N was obtained.

The difference between the theoretical V‐OF value of 15.88 N and the actual measured occlusal force of 15.68 N is 0.2 N, or about 20 gf. This difference is considered to be within the margin of error and the two are considered to be in agreement. This result indicates that the V‐OF derived from the vector sum is the occlusal force. This also indicates that the occlusal force is generated from the balanced contraction force of all masticatory muscles.

The V‐OF is indicated by the bold arrow in Figure 4b. The figure shows that the V‐OF occurs perpendicular to the occlusal plane. This is consistent with the results of parallelism of the occlusal planes of the upper and lower jaws obtained in Experiment 1. In this experiment, we found something even more interesting about the occlusal force, which is that the direction of the occlusal force appears to be toward the center of the Monson sphere (Monson, 1920, 1932).

Next, we found something we consider to be strange in the experiments so far. The contraction force of the posterior muscle bundle of the temporalis muscle was measured as an abnormally large number. This number was larger than the contraction force of the masseter muscle and the occlusal force, even though there was a slight difference between Experiments 1 and 2. The posterior muscle bundle of the human temporalis muscle is the smallest of the three muscle bundles (Kamijo, 1965a), so it is unlikely that it could bear such a large contraction force. Therefore, we consider how the large contraction force of the posterior muscle bundle is borne.

**Figure 5:**
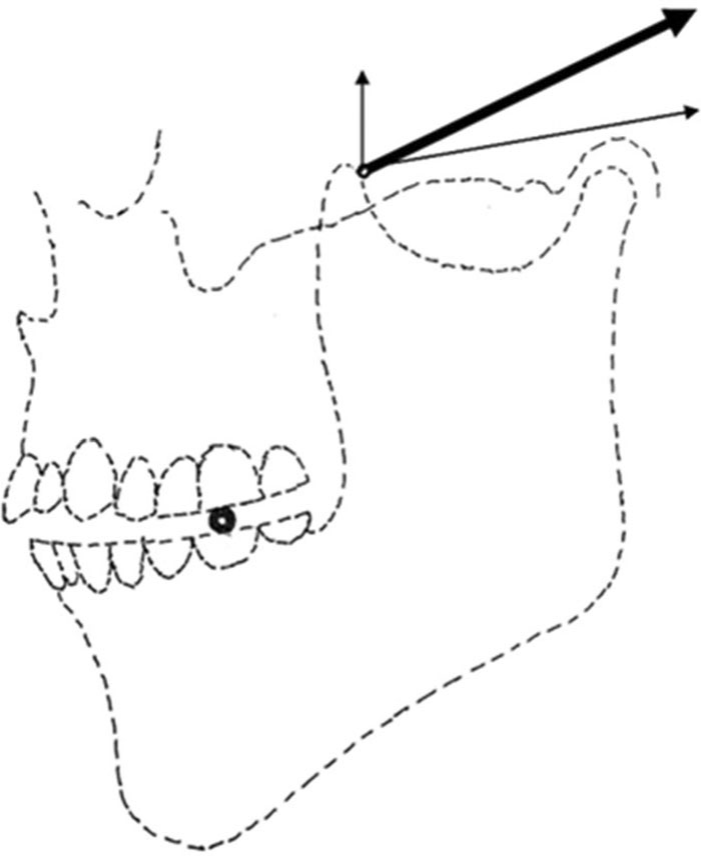
Burden of the large contraction force measured in the posterior muscle bundle of the temporalis muscle.

In the experiment, we did not set up the middle muscle bundle. The reason for this is that we thought that the contraction force of the middle muscle bundle could be analyzed from the contraction force of the anterior and posterior muscle bundles. Therefore, we calculated the sum of the vectors of those two contraction forces. The contraction forces of the anterior and posterior muscle bundles obtained in Experiment 2 are shown by the thin arrow vectors in Figure 5. In addition, the vector obtained from the sum of these two vectors is shown with the bold arrow.

The direction of the vector indicated by the bold arrow is consistent with the direction of the middle muscle bundle (Kamijo, 1965a). In other words, the large contraction force of the posterior muscle bundle measured in the experiments can be borne mainly by the middle muscle bundle. If the experiment is performed with the addition of the middle muscle bundle, the large contraction force of the posterior muscle bundle measured in the experiment is borne by the middle and posterior muscle bundles. This indicates that the large contraction force of the posterior muscle bundle measured in the experiment will be smaller than the occlusal force.

The results of Experiment 2 can be summarized as follows. First, the occlusal force occurred under the balanced contraction force of all the masticatory muscles. Second, the occlusal force was generated as a value greater than the contraction force of all the masticatory muscles. Third, the occlusal force was generated perpendicular to the occlusal plane. Finally, the direction of the occlusal force was assumed to be approximately toward the center of the Monson sphere.

## 4 DISCUSSION

- Unilateral occlusion model and flat occlusal plane
  The experimental apparatus was fabricated as a unilateral occlusion model of the upper and lower posterior teeth, including the temporomandibular joint. We believe that a unilateral occlusion model is occlusally viable because the upper and lower posterior teeth and the temporomandibular joint on one side exist on an almost flat plane (Kamijo, 1965c). Furthermore, it has been reported that the occlusion of the upper and lower posterior teeth on one side is stabilized only by the contractile forces of the masseter, medial pterygoid, and temporalis muscles on the same side (Hasegawa, 1997; Niwa, 2020a).
  The reason why we used a flattened occlusal surface is that there are reports that normal occlusion can be established even with such a flat occlusal surface (Begg, 1977; Niwa, 2020b). These factors led us to believe that a unilateral occlusion model such as the one used in this experiment would be sufficient to achieve the objectives of this study.
  The reason for enlarging the skull to create the occlusion model is to minimize the measurement error as much as possible due to the poor measurement accuracy of the commercially available spring scale. Two types of spring scales were used in the experiment, one with a maximum measurement value of 5 kgf and the other with 2 kgf. The accuracy of each sale is 50 gf for the minimum measurement unit of the 5 kgf scale and 20 gf for the 2 kgf scale. This indicates that the difference of a few tens of grams between the calculated and measured values shown in the two experiments can be considered to be within the measurement error range. Even though the measurement accuracy of the spring scale was poor, we believe that the error could have been reduced by using an enlarged occlusion model.
- Parallelism of the occlusal planes of the upper and lower jaws and the direction of the occlusal force.

From the results of previous experiments, it has been found that when occlusal force is applied to food, the occlusal planes of the upper and lower jaws become parallel, and the occlusal force is generated perpendicularly to the occlusal plane of the upper and lower jaws, and its direction appears to be almost towards the center of the Monson spere (Monson, 1920, 1932).

The parallelism of the occlusal planes indicates that the occlusal force passes from the occlusal point of the mandibular molar in contact with the food to the occlusal point of the maxillary molar. Further developing from this point, we believe that the occlusal planes of the upper and lower jaws during mastication are always in motion while maintaining parallelism. However, we intend to further research its authenticity.

Next, we consider the issue that the occlusal force is directed toward the center of the Monson sphere. To measure the true direction of the occlusal force, we need to measure it in three dimensions. To do this, we need to experiment with the addition of the medial pterygoid muscle. Since the medial pterygoid muscle was not set up in this experiment, the exact three‐dimensional direction of the occlusal force is not known. However, if the direction of the occlusal force is toward the center of the Monson sphere, the direction of the occlusal force and the tooth axis will coincide (Monson, 1920). This coincidence makes a lot of sense in terms of occlusal mechanics. In other words, when a large occlusal force is applied to the tooth when crushing hard food, if the occlusal force passes through the center of the occlusal surface and is aligned with the tooth axis (Okeson, 2003c), there will be no harmful lateral pressure that can shake the tooth during mastication and destroy the bone surrounding the tooth (Niwa, 2020c). In addition, the fact that the occlusal force is directed toward the center of the Monson sphere would mean that the occlusal force would not be concentrated in a specific area of the skull, but would be distributed throughout the skull. This issue will also be the subject of future research.

- Large contraction force measured in the posterior muscle bundle of the temporalis muscle.

In the experiment, a large contraction force was measured in the posterior muscle bundle of the temporalis muscle. It was speculated that the reason for this was that the middle muscle bundle was not included in the experiment. Therefore, to confirm whether or not the middle muscle bundle is really involved in the large contraction force burden of the posterior muscle bundle, we conducted an experiment with the addition of the middle muscle bundle. However, when the contraction force applied to the temporalis muscle was distributed across three muscle bundles, the measured values for each muscle bundle varied from measurement to measurement, and stable values could not be obtained. The reason for this is thought to be that in experiments dealing with a small contraction force such as this one if the contraction force of the temporalis muscle is distributed over three muscle bundles, the contraction force of each muscle bundle will become small and stable values thus could not be obtained.

Although the experiment with the addition of the middle muscle bundle was unsuccessful, the analysis presented earlier clearly shows that the large contraction force of the posterior muscle bundle measured in the experiment is also borne by the middle muscle bundle. It is clear from this that the occlusal force occurs as a value greater than the contraction force of the three muscle bundles of the temporalis muscle.

Next, let's consider the contraction force measured in the masseter muscle. In the experiment, the contraction force of the masseter muscle was large and close to the occlusal force. However, this contraction force is brought about by the contraction force of the masseter muscle and the medial pterygoid muscle. Therefore, when the medial pterygoid muscle was added to the experiment, the contraction force of the masseter muscle would be almost half of the values measured in this experiment. These indicate that the occlusal force is greater than the contraction force of all the masticatory muscles.

- Relationship between occlusal force and the temporomandibular joint

We considered whether the occlusal force generated during masticatory movement causes a load on the temporomandibular joint. When food is present between the upper and lower jaw teeth, the condyle moves anteriorly and downward without contact along the anterior wall of the mandibular fossa. The amount of movement of the condyle is proportional to the size of the food. However, no matter which position of movement the condyle is in, the occlusal force applied to the food does not compress the anterior wall of the mandibular fossa through the condyle. In the central occlusion, which is the terminal position of masticatory movement, the condyle is in the center of the mandibular fossa. This was confirmed in this experiment. In other words, masticatory movement is that during which the TMJ is not subjected to a load from the occlusal force. In the future, we would like to fabricate a three‐dimensional experimental apparatus that can apply a large contraction force so that we can conduct highly accurate experiments.

## 5 CONCLUSIONS

After constructing a unilateral occlusion model and conducting experiments to elucidate the mechanism of occlusal force generation, the following conclusions can be drawn.

1. The occlusal force occurs under the balanced contraction force of all the masticatory muscles.
2. The occlusal force occurs when the occlusal planes of the upper and lower jaws are parallel.
3. The direction of the occlusal force is generated perpendicular to the occlusal plane.
4. The occlusal force appears with a force greater than the contraction force of the individual masticatory muscles.
5. Even if a large occlusal force is applied to the food, the force is not applied to the TMJ.

These can be summarized as follows. In the human masticatory system, even though the contraction force of each masticatory muscle is small, they combine well to generate a large occlusal force, and the generated occlusal force has a mechanism to efficiently act on food without placing a burden on the TMJ.

## AUTHOR CONTRIBUTIONS

All authors made substantial contributions to the study concept or the data analysis or interpretation; drafted the manuscript or revised it critically for important intellectual content; approved the final version of the manuscript to be published; and agreed to be accountable for all aspects of the work.

## CONFLICT OF INTEREST STATEMENT

The authors declare no conflict of interest.

## Data Availability

The data that support the findings of this study are available from the corresponding author upon reasonable request.
